# Predictors of Depressive Symptoms in Autistic Youth—A Longitudinal Study From the Province of Ontario Neurodevelopmental Disorders (POND) Network: Prédicteurs des symptômes dépressifs chez les jeunes autistes—une étude longitudinale du Réseau des troubles neurodéveloppementaux de la province de l’Ontario (réseau POND)

**DOI:** 10.1177/07067437241259925

**Published:** 2024-07-25

**Authors:** Avery Longmore, Evdokia Anagnostou, Stelios Georgiages, Jessica Jones, Elizabeth Kelley, Danielle Baribeau

**Affiliations:** 1 Department of Paediatrics, 7938 University of Toronto, Toronto, ON, Canada; 2 Department of Paediatrics, 7979 The Hospital for Sick Children, Toronto, ON, Canada; 3 Bloorview Research Institute, 37205 Holland Bloorview Kids Rehabilitation Hospital, Toronto, Canada; 4 Department of Psychiatry and Behavioural Neurosciences, 3710 McMaster University, Hamilton, ON, Canada; 5 Department of Psychology, 4257 Queen's University, Kingston, ON, Canada; 6 Department of Psychiatry, University of Toronto, Toronto, ON, Canada; 7 Department of Psychiatry, The Hospital for Sick Children, Toronto, ON, Canada

**Keywords:** autism spectrum disorder, adolescence, child and adolescent psychiatry, depressive disorders, longitudinal study, risk factor

## Abstract

**Objective:**

The objective of this study was to identify longitudinal predictors of depressive symptoms in autistic children and youth.

**Methods:**

Participants were youth with a diagnosis of autism who were part of the Province of Ontario Neurodevelopmental Disorders Network longitudinal substudy. Depressive symptoms were assessed using the child behaviour checklist (CBCL) affective problems subscale. Univariate and multivariable logistic regression models were used to estimate odds ratios (ORs) and 95% confidence intervals (CIs) for the associations between clinical and demographic characteristics at baseline (T1) and clinically elevated depressive symptoms (CEDS) approximately 4 years later (T2).

**Results:**

The mean age of participants (*n* = 75) at T1 was 9.8 years (*SD* = 2.7) and at T2 was 14.1 years (*SD* = 2.8). A total of 37% and 35% of participants had CEDS at T1 and T2, respectively. Additionally, 24% of participants had CEDS at both T1 and T2. T1 characteristics associated with T2 CEDS were: loneliness (OR = 3.0, 95% CI, 1.1 to 8.8), self-harm (OR = 4.0, 95% CI, 1.1 to 16.9), suicidal ideation (OR = 3.9, 95% CI, 1.0 to 16.5), more social and adaptive skills (OR = 0.3, 95% CI, 0.1 to 0.9), elevated restricted and repetitive behaviours (OR = 3.8, 95% CI, 1.3 to 11.6), psychotropic medication use (OR = 3.0, 95% CI, 1.1 to 8.4), attention-deficient/hyperactivity disorder (OR = 2.8, 95% CI, 1.1 to 7.8), and T1 CEDS (OR = 8.8, 95% CI, 3.1 to 27.0) (uncorrected for multiple comparisons). Associations persisted after adjusting for age and intelligence quotient (IQ) differences. Age, sex, IQ, teasing/bullying on the CBCL, family psychiatric history and family income were not associated with T2 CEDS.

**Conclusion:**

Our results highlight both high prevalence and high potential for the persistence of depressive symptoms in autism and emphasize the importance of early support to address loneliness and social participation.

## Plain Language Summary Title

Study assessing risk factors for depression in autistic youth

**Plain Language Summary**

**Objective:**

The goal of this study was to find risk factors for depression in autistic youth.

**Methods:**

The study included autistic youth who were part of the Province of Ontario Neurodevelopmental Disorders Network. Symptoms of depression were identified using mental health surveys and screening tools completed by parents. We studied 75 youth over two time points, to understand what factors might predict greater depression risk.

**Results:**

The average age of our study population at the first visit was 10 years old, and 14 years old at the second visit. Our study found that 37% of participants had elevated symptoms of depression at the first visit, and 35% at the second visit. Factors associated with future depressive symptoms included: loneliness, self-harm, suicidal ideation, high levels of restrictive/repetitive behaviours, depressive symptoms at the first visit, and ADHD. Factors that protected against depressive symptoms included high levels of social skills.

**Conclusion:**

Our results show high levels of depressive symptoms among autistic youth, and the potential for this to persist over time in this population. Our findings emphasize the importance of early supports to address loneliness and social participation.

## Résumé

**Objectif:**

L’objectif de la présente étude était d’identifier les prédicteurs longitudinaux des symptômes dépressifs chez les enfants et les jeunes autistes.

**Méthodes:**

Les participants étaient des jeunes ayant reçu un diagnostic d’autisme qui participaient à la sous-étude longitudinale du Réseau des troubles neurodéveloppementaux de la province de l’Ontario (POND). Les symptômes dépressifs étaient évalués à l’aide de la sous-échelle des problèmes affectifs de la liste de contrôle du comportement de l'enfant (LCCE). Des modèles de régression logistique univariée et multivariable ont servi à estimer les rapports de cotes (RC) et les intervalles de confiance (IC) à 95% ont été utilisés pour les associations entre les caractéristiques cliniques et démographiques au début (T1) et les symptômes dépressifs cliniquement élevés (SDCE) approximativement 4 ans plus tard (T2).

**Résultats:**

L’âge moyen des participants (n = 75) à T1 était de 9,8 ans (ET = 2,7) et à T2 il était de 14,1 ans (ET = 2,8). Un total de 37% et de 35% des participants avaient des SDCE à T1 et T2, respectivement. En outre, 24% des participants avaient des SDCE autant à T1 qu’à T2. Les caractéristiques à T1 associées aux SDCE à T2 étaient la solitude (RC = 3,0, IC à 95% 1,1 à 8,8), l’automutilation (4,0, 1,1 à 16,9), l’idéation suicidaire (3,9, 1,0 à 16,5), les compétences sociales et adaptatives (0,3, 0,1 à 0,9), des comportements restreints et répétitifs élevés (3,8, 1,3 à 11,6), l’usage de médication psychotrope (3,0, 1,1 à 8,4), le TADAH (2,8, 1,1 à 7,8), et les SDCE à T1 (8,8, 3,1 à 27,0) (non corrigé pour des comparaisons multiples). Les associations persistaient après ajustement pour l’âge et les différences de QI. L’âge, le sexe, le QI, la taquinerie/intimidation sur la LCCE, les antécédents psychiatriques familiaux et le revenu familial n’étaient pas associés aux SDCE à T2.

**Conclusion:**

Nos résultats révèlent à la fois la prévalence élevée et le potentiel élevé de la persistance des symptômes dépressifs dans l’autisme et soulignent l’importance de soutiens précoces pour lutter contre la solitude et aborder la participation sociale.

## Background

Depressive disorders are the leading cause of disability worldwide.^1^ In Canada, adolescents and young adults have the highest rate of mood disorders, with 11% experiencing depression in their lifetime.^2^ Mood disorders are also one of the most common comorbidities experienced by autistic youth. A recent meta-analysis found that autistic individuals are 4 times more likely to experience depression as compared to nonautistic individuals,^3^ and autistic youth are also more likely to attempt suicide.^4^ Furthermore, depression has been shown to be one of the major predictors of quality of life and overall functioning in autism.^5^ However, depression may be harder to recognize or diagnose in autistic individuals, given overlap with or overshadowing by autistic traits, limited research on manifestations of depression in autism, and few validated depression screening measures for this population.^6^

There is also limited evidence in the literature regarding risk factors for depression in autistic youth and some of the findings are inconsistent.^7^ Like nonautistic youth, the prevalence of depression may increase with age, especially during adolescence.^6–9^ Most studies of autistic youth do not find sex-based differences in rates of depression, which contrasts with nonautistic youth where female sex is a risk factor.^3,6,7,9^ Higher intelligence quotient (IQ), the timing of autism diagnosis, and higher levels of autistic traits, including higher levels of restrictive and repetitive behaviours, have also been associated with depression in autistic individuals in cross-sectional samples.^3,7–13^ Finally, negative social experiences and bullying have been shown to contribute to depressive symptoms, while positive social experiences have been shown to be a protective factor.^7,14,15^

The studies examining risk factors for depression in autism have mostly included cross-sectional cohorts, with limited longitudinal data.^8–11,15^ Existing longitudinal studies have focused on autistic adults,^16^ or have had small sample sizes.^14,17^ Using a longitudinal sample of youth from the Province of Ontario Neurodevelopmental Disorders (POND) Network, the aim of this study was to identify individual, family, and environmental factors associated with increased risk for future depressive symptoms in autistic youth.

## Study Methods

### Participants and Study Design

Participants were recruited through the POND Network, which is a multicentre collaborative research program that includes 11 sites across Ontario, Canada. The general inclusion criteria for POND are age 0–21 years, a diagnosis of 1 or more neurodevelopmental disorders, and birth after 35 weeks gestation. For this analysis, we restricted the sample to youth with a primary diagnosis of autism and who had completed 1 or more follow-up assessments through the POND longitudinal substudy (T1 = baseline and T2 = first follow-up visit). Participant data were excluded if they had not completed at least two instances of the Child Behavior Checklist (CBCL), as this was the longitudinal mental health outcome for the study. This effectively restricted the sample to participants ages 6–18 years given the intended age range and application of the measure in the study protocol. Autism diagnoses were verified upon study entry using gold standard assessments including the Autism Diagnostic Observation Schedule-Second Edition and the Autism Diagnostic Interview-Revised (ADI-R).^18,19^

### Ethics

This study was approved by the Holland Bloorview Children's Rehabilitation Hospital Research Ethics Board, and all POND sites received local Research Ethics Board approval. Written informed consent and verbal assent were obtained from all participants and/or their primary caregivers.

### Measures

#### Demographic and Medical Data

Age (in years), biological sex, family income (with <$75,000/year household income considered low income), timing of autism diagnosis (age in years), psychotropic medication use at intake and family psychiatric history (any psychiatric condition in first degree relatives) were captured on the medical and demographics intake forms.

#### Developmental Measures

Cognitive abilities were assessed using standardized IQ assessments (Stanford-Binet Intelligence Scales, the Wechsler Abbreviated Scales of Intelligence, Leiter International Performance Scale, Mullen Scales of Early Learning, Wechsler Intelligence Scale for Children, or Wechsler Preschool & Primary Scale of Intelligence).^20–25^ Levels of autistic traits were assessed through the Social Communication Questionnaire (SCQ) and the ADI-R. The SCQ is a brief parent-report measure on autistic traits and behaviours that includes 40 items.^26^ The ADI-R is a standardized parent interview widely used in the diagnosis of autism, which includes a scoring algorithm with subscales in social, communication, and repetitive behaviour domains.^18^ We used the ADI-R restricted and repetitive behaviour domain total score, and the social domain total score (both using the total sum from the diagnostic algorithm). Adaptive behaviours were assessed through the Adaptive Behaviour Assessment System (ABAS), including the general adaptive composite standard score and social composite score for ages 5–21. The ABAS is a parent rating scale that assesses various adaptive skills including communication, home living, self-care, and social interactions across the lifespan.^27^ Co-occurring attention-deficit/hyperactivity disorder (ADHD) symptoms were identified using the Strengths and Weaknesses of ADHD Symptoms and Normal Behavior Scale (SWAN).^28^ Participant's level of communication was identified by the ADI-R item 30 “overall level of language,” which was categorized as verbal or nonverbal/minimally speaking.

#### Other Predictors of Interest

Additional a priori predictors of interest from review of the literature were selected from measures available. In addition to the demographic and clinical characteristics above, specific items from the CBCL were used as markers for loneliness, teasing/bullying, self-harm, and suicidality as these were not captured in other measures. We note that two of these items (self-harm and suicidality) are items included within the CBCL affective problems subscale items, while two others are not (loneliness and teasing/bullying).

### Outcomes

Mental health outcomes were assessed using the CBCL 6–18 parent report measure. The CBCL is a commonly used tool to screen for emotional/behavioural concerns in children. Recent studies have shown psychometric support for the use of the CBCL Affective Problems subscale to screen for depression in autism.^29–31^ Upon analysis of a similar patient population to the present study (autistic youth with a mean age of 11 years), Magyar and Pandolfi^29^ found that the CBCL affective problems subscale correlated with diagnoses of depression on The Kiddie Schedule for Affective Disorders and Schizophrenia, a well-known semistructured diagnostic interview based on the Diagnostic and Statistical Manual of Mental Disorders (DMS) criteria for mood disorders which are broadly utilized within clinical research.^32,33^ They also found that with a raw score cut-off of 6.5, the CBCL affective problems subscale demonstrated good diagnostic accuracy for depression, with a sensitivity of 95%, a specificity of 74%, and an area under the curve (AUC) = 0.88.^29^ Thus, in the present study, the mental health outcome of “clinically elevated depressive symptoms” (CEDS) was assessed using a cut-point total raw score of >6.5 on the CBCL affective problems subscale.

### Statistical Analyses

We reported descriptive statistics for the demographics and clinical characteristics of the study population. A univariate logistic regression model was used to examine predictor variables at T1 and their association with CEDS at T2. These predictors were chosen a priori based on a literature review of similar studies suggesting an association with depression in autism. This model generated crude odds ratios (ORs) and 95% confidence intervals (95% CIs). We also calculated adjusted ORs (aORs), including a covariate for age at T2 (given a known association between depressive symptoms and adolescence in the general population) and for IQ (given prior research suggesting an association with IQ,^3,9^ and potential additional complexity in assessing mood symptoms as well as a need for further validation of CBCL subscales in those with intellectual disability).^34,35^

Several continuous measures were dichotomized to better visualize effects in the forest plot, for clinical interpretability, and to capture potential nonlinear associations. For standardized scores (IQ, ABAS) cut-offs of 70 were used. For the SCQ and the ADI-R restrictive and repetitive behaviour subscale, we used previously published cut-offs (total scores of 15 and 7, respectively).^26,36^ For the ADI-R social domain, we considered total scores above 23 (top 25% of this sample) to be “elevated.”

As a sensitivity analysis, we used a multivariable logistic regression model to estimate aORs and 95% CI for predictors of interest from prior models while also accounting for baseline (T1) levels of depressive symptoms on the CBCL.

All models were examined for influential outliers (confirming standardized residuals of all outliers <3), and multivariable models were examined for multicollinearity (all variance inflation factors (VIF) < 4). Model discrimination was examined through receiver operating curve characteristics comparing predicted outcomes to true outcomes, with an area under the curve (AUC)  > 0.8 considered adequate discrimination. The Hosmer–Lemeshow goodness of fit test was used to assess multivariable model fit.

As an exploratory analysis, we compared baseline demographic characteristics and clinical predictors between participants with and without persistent depressive symptoms across both T1 and T2. The groups were compared using *t*-tests, the Wilcoxon rank-sum test, or Pearson's chi-squared test with the significant level set at 0.05.

## Results

**Figure 1.**
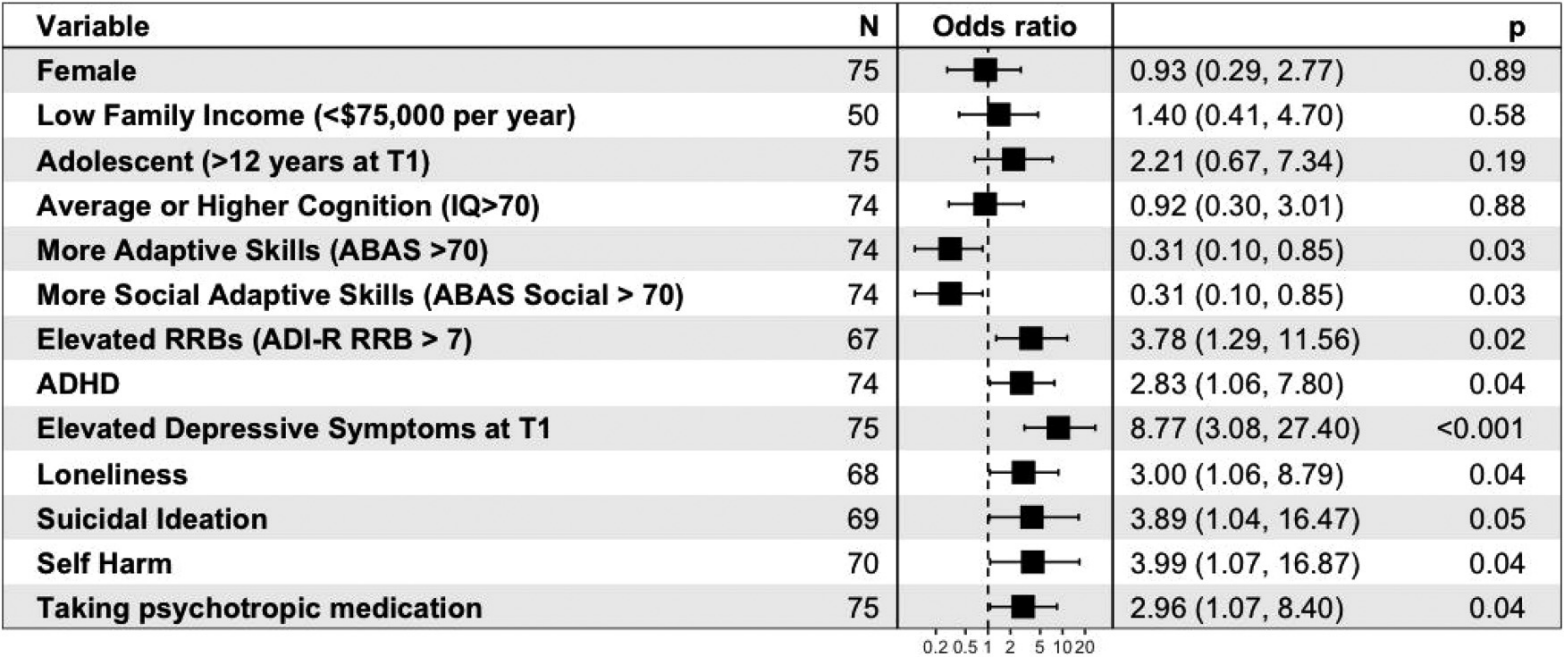
Summary of univariate predictors of longitudinal depressive symptoms in autism. Variables at T1 are listed, with their calculated odds ratios for CEDS at T2, and 95% CI in brackets. *P*-values are also listed for each univariate measure. Cut-points used for various measures are as follows: adaptive skills: ABAS Global Composite Score > 70; level of autistic traits: SCQ total score > 15; social skills: ABAS social composite score > 70; level of restrictive and repetitive behaviours: ADI-R restrictive and repetitive behaviour domain total score > 7; elevated symptoms of depression at T1: CBCL affective problems subscale raw score > 6.5; loneliness, suicidal ideation, and self-harm: presence of symptoms indicated on individual CBCL items.

*Note*. ABAS* *=* *adaptive behaviour assessment system; CEDS* *=* *clinically elevated depressive symptoms; SCQ* *=* *social communication questionnaire; ADI-R* *=* *Autism Diagnostic Interview-Revised; CBCL* *=* *Child Behavior Checklist; T1* *=* *time point 1, baseline; T2* *=* *time point 2, follow-up.

**Figure 2.**
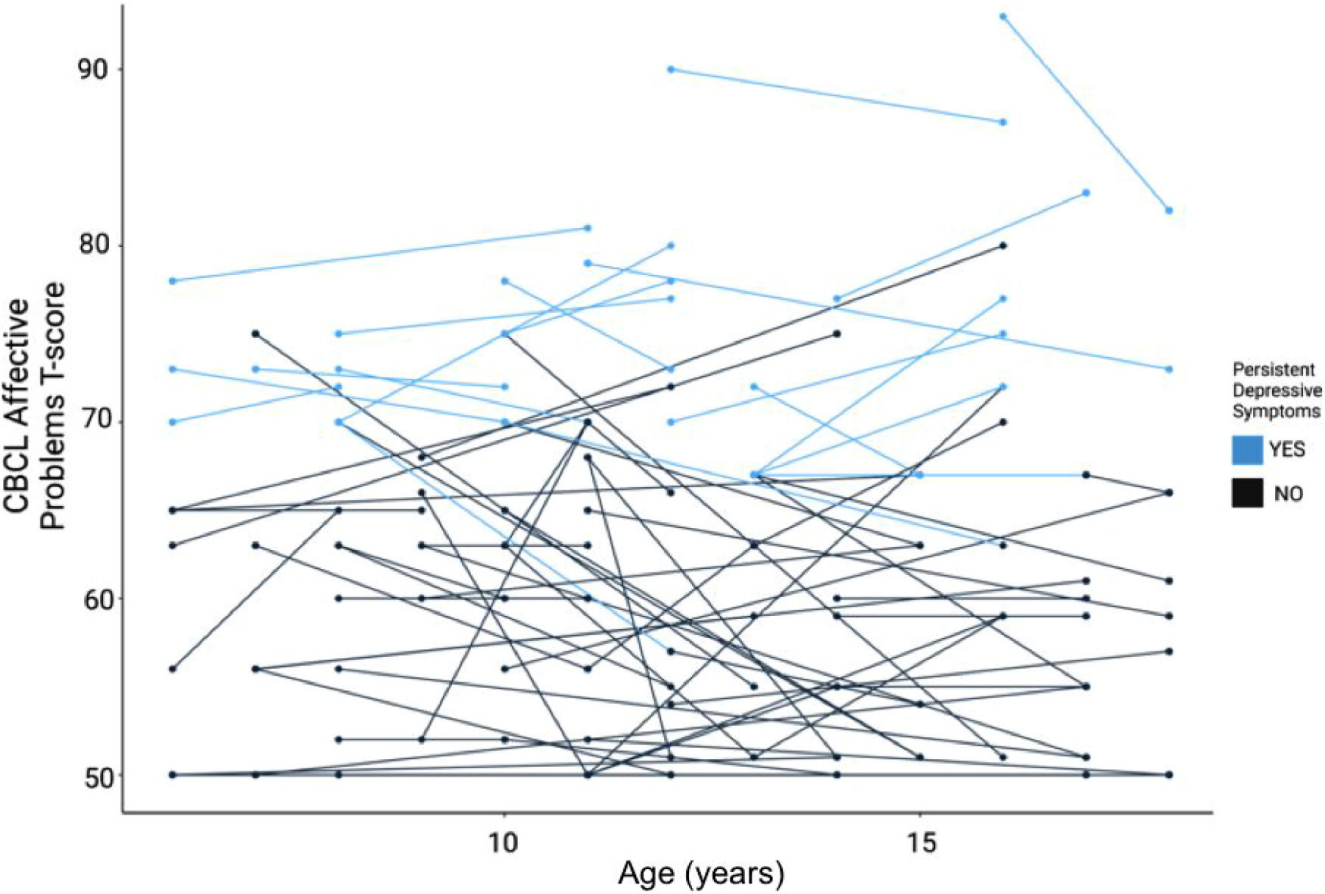
Depressive symptoms categorized by age and persistence, by the study participant. The *y*-axis denotes the CBCL affective problems T-score, with levels above 60 indicating clinically elevated depressive symptoms relative to general population samples used to derive this measure. The *x*-axis shows the participant's age at each longitudinal follow-up. Evidence of persistent depressive symptoms (defined as a total CBCL raw score above 6.5 at both T1 and T2) is highlighted in a lighter shade.

*Note*. CBCL* *=* *Child Behavior Checklist; T1* *=* *time point 1, baseline; T2* *=* *time point 2, follow-up.

**Demographics and Clinical Characteristics**: A total of 75 youth with a primary diagnosis of autism met the inclusion criteria for this analysis (Table 1). The average age of participants was 9.8 years (*SD* = 2.7) at T1, and the average amount of time between T1 and T2 was 4.3 years (*SD* = 2.2). Most participants were diagnosed with autism between the ages of 2–5 years (*N* = 41, 55%). Most participants identified as male (*N* = 57, 76%), and white (*N* = 40, 53%). With respect to mental health outcomes, 37.3% of participants had CEDS at T1 and 34.7% at T2 (Table 2). The majority of participants (69%) with CEDS at T2, also had CEDS at T1. Demographic data and clinical characteristics of the study population are reported in Table 1. Longitudinal mental health outcomes are highlighted in Figures 1 and 2, and Table 2. Correlations between measures are presented in Supplemental Table S1.

**Table 1.** Demographic and Clinical Characteristics.

	Baseline (T1) (*n* = 75)
**Age**	
Mean (*SD*)	9.8 (2.7)
Median [Min, Max]	10.0 [6.0, 17.0]
**Sex**	
Female	18 (24.0%)
**IQ**	
IQ mean (*SD*)	90.9 (26.9)
Median [Min, Max]	93.0 [40.0, 142]
**Family income**	
<$74,999	17 (22.7%)
Prefer not to answer or missing	24 (32.0%)
**Age of ASD diagnosis**	
Mean (*SD*)	4.9 (2.8)
Median [Min, Max]	4.0 [0, 13.0]
**Communication**	
Minimally speaking	6 (8.0%)
**Developmental assessments**	
SCQ total score, mean (*SD*)	20.1 (7.0)
Adaptive behaviour global composite, mean (*SD*)	67.8 (19.1)
Adaptive behaviour social domain, mean (*SD*)	72.8 (16.6)
ADI-R RRB total, mean (*SD*)	6.4 (2.8)
ADI-R social domain total, mean (*SD*)	21.1 (4.9)
**Number of psychiatric medications**	
0	52 (69.3%)
1	10 (13.3%)
2	9 (12.0%)
>2	4 (5.4%)
Any	23 (30.6%)
**Family history of psychiatric disorder**	
Yes	22 (29.3%)

*Note*: *SD *=* *standard deviation; ASD* *=* *autism spectrum disorder; SCQ* *=* *Social Communication Questionnaire; IQ* *=* *intelligence quotient; ADI-R* *=* *Autism Diagnostic Interview-Revised; ADI-R RRB* *=* *ADI-R Restricted and Repetitive Behaviour Subscale Score; <74,999 was used as the low-income cut-off for this study, capturing the lowest four income quintiles.

**Table 2.** Depressive Symptoms on the CBCL Across Two Time Points.

	Time 1	Time 2
**Age**		
Mean (*SD*)	9.8 (2.7)	14.1 (2.8)
Median [Min, Max]	10.0 [6.0, 17.0]	15.0 [8.0, 18.0]
**CEDS**		
Yes	28 (37.3%)	26 (34.7%)
**CBCL affective total score**		
Mean (*SD*)	5.3 (4.3)	5.3 (4.6)
Median [Min, Max]	5.0 [0, 22.0]	4.0 [0, 18.0]
**CBCL affective T-score**		
Mean (*SD*)	63.7 (9.6)	62.6 (10.1)
Median [Min, Max]	63.0 [50.0, 93.0]	61.0 [50.0, 87.0]

*Note*: SD* *=* *standard deviation; CEDS* *=* *clinically elevated depressive symptoms; CBCL* *=* *Child Behavior Checklist**.** CEDS defined as CBCL affective problems subscale total score > 6.5.^29^

**Longitudinal Association Between T1 Predictors and T2 CEDS**: Results of the univariate (crude) logistic regression models and multivariate (adjusted for T2 age and IQ) logistic regression models examining the association between baseline (T1) demographic, developmental and clinical variables and follow-up (T2) CEDS are reported in Table 3, and key findings are summarized in Figure 1. For multivariate models, there was no evidence of influential outliers (all standardized residuals <3), or multicollinearity (all VIF < 4); models showed adequate discrimination (AUC > 0.8) and adequate fit (Hosmer–Lemeshow *p* > 0.1).

**Table 3.** Longitudinal Predictors of Elevated Depressive Symptoms in Autistic Children and Youth.

	Univariate		Adjusted for IQ and Age at T2	
Predictor	OR (95% CI)	*P*-value	OR (95% CI)	*P*-value
**Demographic**				
Low family income (<$75,000/year)	1.40 (0.41 to 4.70)	0.58	1.48 (0.41 to 5.29)	0.55
Age at T1	1.01 (0.84 to 1.21)	0.94	1.13 (0.87 to 1.47)	0.36
Age at T2	0.93 (0.79 to 1.11)	0.43		
Female sex	0.93 (0.29 to 2.77)	0.89	0.75 (0.23 to 2.46)	0.63
Adolescent (>12 years) at T1	2.21 (0.67 to 7.34)	0.19	2.32 (0.71 to 7.62)	0.17
**Developmental**				
IQ (continuous)	0.99 (0.98 to 1.01)	0.80	0.99 (0.97 to 1.01)	0.60
IQ > 70	0.92 (0.30 to 3.01)	0.88	0.84 (0.27 to 2.7)	0.77
General adaptive skills (continuous)	0.97 (0.94 to 0.99)	**0**.**04**	0.97 (0.93 to 0.99)	**0**.**04**
More adaptive skills (ABAS ≥ 70)	0.31 (0.10 to 0.85)	**0**.**03**	0.32 (0.11 to 0.98)	**0**.**05**
SCQ total (continuous)	1.03 (0.96 to 1.11)	0.36	1.03 (0.95 to 1.11)	0.44
More autistic traits (SCQ >15)	2.02 (0.50 to 8.10)	0.32	1.88 (0.46 to 7.7)	0.38
ABAS social composite (continuous)	0.97 (0.93 to 0.99)	**0**.**05**	0.96 (0.93 to 1.00)	0.06
More adaptive social skills (ABAS > 70)	0.31 (0.10 to 0.85)	**0**.**03**	0.33 (0.11 to 0.98)	**0**.**05**
ADI-R RRB (continuous)	1.28 (1.04 to 1.56)	**0**.**02**	1.28 (1.05 to 1.57)	**0**.**02**
High ADI-R RRB (>7)	3.78 (1.29 to 11.56)	**0**.**02**	3.85 (1.27 to 11.65)	**0**.**02**
ADI-R social domain (continuous)	0.98 (0.88 to 1.08)	0.61	0.98 (0.88 to 1.09)	0.72
High ADI-R social domain (>23)	1.60 (0.54 to 1.48)	0.41	1.54 (0.49 to 4.74)	0.46
ADHD	2.83 (1.06 to 7.80)	**0**.**04**	2.70 (0.97 to 7.60)	0.06
**Mental health**				
Elevated depressive symptoms T1	8.77 (3.08 to 27.40)	**<0**.**001**	9.68 (3.05 to 30.67)	**<0**.**001**
Loneliness	3.00 (1.06 to 8.79)	**0**.**04**	3.63 (1.18 to 11.11)	**0**.**02**
Suicidal ideation	3.89 (1.04 to 16.47)	**0**.**05**	4.49 (1.24 to 16.28)	**0**.**02**
Self-harm behaviour	3.99 (1.07 to 16.86)	**0**.**04**	5.91 (1.33 to 26.11)	**0**.**02**
Teasing/bullying	1.48 (0.55 to 3.96)	0.43	1.62 (0.59 to 4.48)	0.35
Psychotropic medications (any)	2.96 (1.07 to 8.40)	**0**.**04**	3.47 (1.20 to 10.04)	**0**.**02**
Family psychiatric history	1.11 (0.39 to 3.14)	0.84	1.33 (0.45 to 3.90)	0.61

*Note*. T1* *=* *time point 1, baseline; T2* *=* *time point 2, follow-up; OR* *=* *odds ratio; CI* *=* *confidence interval; SCQ* *=* *Social Communication Questionnaire; ABAS* *=* *Adaptive Behaviour Assessment System; ADI-R* *=* *Autism Diagnostic Interview-Revised; ADI-R RRB* *=* *ADI-R Restricted and Repetitive Behaviour Subscale Score; ADHD* *=* *attention-deficit/hyperactivity disorder.

Loneliness, self-harm, and suicidal ideation were assessed on the Child Behavior Checklist.

General adaptive and social skills were assessed using the Adaptive Behaviour Assessment System.

Restricted/repetitive behaviours were assessed on the ADI-R.

Family psychiatric history and medication use refers to any history of mental health conditions in first-degree relatives and any psychotropic medication use by the participant at T1. Bolded values = *p* < 0.05.

**Demographic Variables**: Participant age, biological sex, and income were not significantly associated with CEDS at T2, in either crude or adjusted models. *Developmental variables*: Participants with more general adaptive skills (ABAS global composite score), as well as more social adaptive skills (ABAS social composite), had significantly decreased odds of experiencing CEDS at T2. Participants with higher scores on the ADI-R repetitive behaviour domain had an increased risk of CEDS at T2. Overall level of autistic traits (SCQ), autistic traits in the social domain (ADI-R social total) and IQ were not significantly associated with odds of CEDS. Co-occurring ADHD was associated with CEDS in the univariate model. For continuous measures that were dichotomized, patterns of association and significance were similar to continuous models. Scatter plots for specific variables of interest are presented in supplemental materials to visualize associations.

**Other Clinical and Mental Health Variables of Interest**: CEDS at T1, loneliness, history of self-harm, and a history of taking psychotropic medication all had significantly increased odds of CEDS at T2, across univariate and multivariate models. History of bullying/teasing and family psychiatric history were not significantly associated with CEDS at T2.

**Sensitivity Analyses**: Significant predictors of interest from crude and adjusted models were further examined also covarying for baseline presence or absence of CEDS at T1 (Supplemental Table S2). Associations or trends persisted for loneliness (aOR 3.6, 95% CI, 0.9 to 12.9), more adaptive skills (aOR 0.28, 95% CI, 0.08 to 1.0), more social adaptive skills (aOR 0.28, 95% CI, 0.08 to 1.0), and high levels of restricted/repetitive behaviours (aOR 3.43, 95% CI, 1.0 to 11.9), but not self-harm suicidal ideation, ADHD, or psychotropic medication use.

We also repeated the univariate and multivariable models using a CBCL affective problems T-score of 65 or greater (normed relative to a nonautistic population) to identify those with CEDS; the results did not change.

**Exploratory Analysis—Subgroup with Persistent Depressive Symptoms**: Our analyses found that 34.7% of study participants had CEDS at T2, and 69% of these individuals also had CEDS at T1 (Figure 2). As an exploratory analysis, we designated individuals with CEDS at both T1 and T2 as the “persistent depressive symptoms” (PDS) subgroup. The PDS subgroup included *N* = 18 participants, 24% of the total study population. The PDS group had higher levels of depressive symptoms at T1 (*p* < 0.001), were more likely to be taking psychotropic medication (*p* = 0.009) and were more likely to have ADHD (*p* = 0.04) (Supplemental Table S3).

## Discussion

Our study aimed to identify early predictors of depressive symptoms among autistic youth using longitudinal data collected by the POND network across Ontario, Canada. Our results demonstrated (a) that elevated depressive symptoms are prevalent among autistic youth, (b) that autistic youth may have elevated rates of persistent depressive symptoms, and (c) that history of loneliness, self-harm behaviour, level of adaptive skills, level of repetitive behaviours, suicidal ideation, psychotropic medication use, and ADHD may be potential early predictors of risk for depressive symptoms in autistic youth, although only baseline depressive symptoms remained a significant predictor of future depressive symptoms after a correction for multiple testing.

To our knowledge, the present study is one of few to analyze longitudinal depressive symptoms in autistic youth during adolescence. Neither age nor sex were significantly predictive of CEDS in this sample, nor was age correlated with depressive symptomatology overall. Most prior studies of depression in autistic youth do not find significant sex differences,^6,7,9,10^ although results have been mixed with respect to age.^7–9,37^ While depressive symptoms have been known to evolve during adolescence, and have been linked to the timing of puberty,^6,38^ the median age of onset of depressive disorders is not until 30 years.^39^ Given that the average age in our study was 10 to 14 years, further follow-up into adulthood may be needed to reveal age effects on depressive symptoms in autism. However, consistent with our findings, previous literature has demonstrated higher levels of depressive symptoms in autistic early adolescents (age 10–13) when compared to nonautistic early adolescents.^6^ This may suggest symptoms of depression present earlier in autistic youth as compared to nonautistic peers. Nonetheless, our data are consistent with others emphasizing the importance of early screening for mood disorders in autism.^6^

Regarding developmental differences, we did not find a significant association between IQ and elevated depressive symptoms, in contrast to some prior studies,^3,7,9,10^ and related to theories that autistic youth with higher IQs may be more affected by social differences and exclusion.^7,9,10^ Our population had a wide range of IQ levels from 40 to 142, and 17 participants (22.6%) had IQ levels below 70. IQ levels examined in previous studies have been variable^7,9,10^ and may have some impact on the differences reported in the literature. Findings with respect to IQ should be interpreted cautiously in light of concerns with the reliability and validity of parent report measures to identify depression in youth with limited verbal communication or intellectual disabilities.^35^

Higher scores on the ADI-R restrictive and repetitive behaviour domain were associated with increased risk of CEDS, as has been shown in prior cross-sectional samples.^7,11–13^ Higher levels of general and social adaptive functioning were potentially protective for CEDS, yet autistic social traits (on the SCQ and ADI-R) were not associated with CEDS, and loneliness was a risk factor that persisted across multiple sensitivity analyses. Our analyses highlight the complexity and nuance surrounding the role of social abilities, social drive, and social experiences on mood in autism. Our findings could suggest that youth with unmet social needs (i.e., loneliness) may be more vulnerable to mood symptoms, irrespective of social abilities or social differences, and that social participation may be protective. Consistent with this, a recent meta-analysis found both a higher prevalence of loneliness and a significant pooled correlation between loneliness and depression in autistic individuals.^40^ Our work provides longitudinal evidence of this association and highlights the importance of challenging previous notions about social motivation in autism,^41,42^ while considering inclusive social environments for autistic youth as part of the foundation for mental health supports. Future work is needed to disentangle these factors to help identify points of intervention.

The co-occurrence of ADHD symptoms and autism has been well described in the literature.^43^ Our study found that clinically elevated scores on the SWAN ADHD assessment were significantly associated with CEDS at T2. This is in keeping with the results of the National Survey of Children's Health 2016–2019 where autistic adolescents with co-occurring ADHD had the highest prevalence of depression.^44^ Higher levels of ADHD symptoms in autistic youth may also be associated with a higher prevalence of comorbid psychiatric diagnoses broadly,^45^ in keeping with concepts of transdiagnostic neuropsychiatric vulnerability.

Throughout the two time points in our study, CEDS was highly prevalent (35%–37%), in keeping with prevalence estimates from previous studies.^2–4^ Our results also suggest that autistic youth may be at risk of experiencing persistent depressive symptoms. Not only was T1 CEDS associated with an approximately 9-fold increased odds of CEDS later in adolescence, but 69% of participants with CEDS at T1 also had CEDS at T2, on average 4 years later (24% of all participants). Hollocks et al.^17^ similarly found that symptoms of an emotional disorder significantly persisted from childhood to adolescence in autistic youth (OR = 8.38; 95% CI, 1.4 to 50.4; *p* = 0.02). In comparison, studies evaluating depression in nonautistic youth have suggested that children who experience symptoms of a mood disorder are only 3 times more likely to develop a mental health condition in adolescence, with the overall prevalence of persistent depressive disorder in children ranging from 0.6% to 4.6%.^46,47^ Mental health conditions have also been shown to persist across adulthood in a longitudinal study with autistic adults aged 19–80,^48^ and remain stable on 12-month follow-up in other studies.^16^ Symptoms of depression may contribute most in predicting quality of life outcomes for transition-aged autistic youth,^5^ and significantly impact self-reported quality of life in autistic adults.^48^ Replication is needed to confirm findings but support the need for early and targeted interventions for mood disorders in autism, given the high potential prevalence, and potential for persistence over time.

Our study has several limitations. First, given our sample size, we were underpowered to detect small effects or to conduct multivariable models with more than 3 predictors. We note that with a sample size of 75, and an outcome affecting 37% of our sample, we were powered to detect moderate to large effect sizes only (Cohen's *d* = 0.7, alpha = 0.05, power = 0.80). We also conducted multiple comparisons, with unadjusted *p*-values reported to convey trends. Even when considering approximately 20 hypotheses tested, the probability of identifying a significant association at *p* < 0.05 by chance alone is high (64%). Using a Dunn–Šidák correction for multiple comparisons, only associations with a *p*-value < 0.003, (effectively baseline depressive symptoms but no other predictors), would remain significant. Further, the assumption of the persistence of depressive symptoms between time points may not apply to all individuals (e.g., they may have had two depressive periods). The ABAS has historically been used to examine social ability in autistic youth with lower IQ, and while there is evidence for its utility in assessing adaptive functioning in autistic youth with higher IQ,^49^ it may be less sensitive in this population than other measures with respect to identifying social and adaptive challenges.^50^ We also used the CBCL affective problems subscale as a surrogate marker for depression. While there have been studies to demonstrate its validity in assessing depressive symptoms in autism using a sample with similar demographics to the current study, it is not equivalent to a clinical diagnosis of major depressive disorder, and we may be missing effects by utilizing the same cut point at two different age groups (e.g., one being preadolescence, and one during adolescence). Additionally, there is some evidence that subscale-level data of the CBCL may not be as valid as item-level data in intellectually heterogeneous samples of autistic youth.^50^ Future evaluation of the validity of CBCL cut-points during adolescence in autistic youth, as well as for autistic youth with intellectual disabilities is needed. Further, mental health outcome data were provided by parent reports, which may not necessarily capture youth experiences of depressive symptoms. We also did not examine co-occurring psychiatric disorders or symptoms (e.g., anxiety). Future work incorporating multimethod and multi-informant measures to contextualize these results and expanding analyses beyond the age of 18 years to incorporate the experiences of emerging autistic adults will be essential.

Overall, our study is one of the first to specifically examine longitudinal predictors of depressive symptoms in autistic youth. We found that CEDS are both prevalent and potentially persistent in autism. High levels of restrictive and repetitive behaviours, loneliness, self-injury, suicidal ideation, psychotropic medication use, and ADHD were all associated with an increased risk of elevated depressive symptoms, while high levels of social and adaptive functioning could be protective. This study demonstrates the important relationship between social connection and emotional well-being in autism and justifies research into depression screening, as well as interventions to treat or prevent depression in autism by targeting social adaptive functioning/inclusivity and loneliness.

## Supplemental Material

sj-docx-1-cpa-10.1177_07067437241259925 - Supplemental material for Predictors of Depressive Symptoms in Autistic Youth—A Longitudinal Study From the Province of Ontario Neurodevelopmental Disorders (POND) Network: Prédicteurs des symptômes dépressifs chez les jeunes autistes—une étude longitudinale du Réseau des troubles neurodéveloppementaux de la province de l’Ontario (réseau POND) Supplemental material, sj-docx-1-cpa-10.1177_07067437241259925 for Predictors of Depressive Symptoms in Autistic Youth—A Longitudinal Study From the Province of Ontario Neurodevelopmental Disorders (POND) Network: Prédicteurs des symptômes dépressifs chez les jeunes autistes—une étude longitudinale du Réseau des troubles neurodéveloppementaux de la province de l’Ontario (réseau POND) by Avery Longmore, Evdokia Anagnostou, Stelios Georgiages, Jessica Jones, Elizabeth Kelley and Danielle Baribeau in The Canadian Journal of Psychiatry
