# Supplementary material for: Predictors of Depressive Symptoms in Autistic Youth—A Longitudinal Study From the Province of Ontario Neurodevelopmental Disorders (POND) Network: Prédicteurs des symptômes dépressifs chez les jeunes autistes—une étude longitudinale du Réseau des troubles neurodéveloppementaux de la province de l’Ontario (réseau POND)
Source: Can J Psychiatry. 2024 Jul 25;70(5):372–81. doi: 10.1177/07067437241259925 (PMC11572051; doi:10.1177/07067437241259925)
Supplement: sj-docx-1-cpa-10.1177_07067437241259925 - Supplemental material for Predictors of Depressive Symptoms in Autistic Youth—A Longitudinal Study From the Province of Ontario Neurodevelopmental Disorders (POND) Network: Prédicteurs des symptômes dépressifs chez les jeunes autistes—une étude longitudinale [file sj-docx-1-cpa-10.1177_07067437241259925.docx]

Supplemental Materials

**Predictors of Depression in Autistic Youth – A Longitudinal Study from the Province of Ontario Neurodevelopmental Disorders (POND) Network**

**Supplemental Table 1: Correlation matrix of continuous variables**

|  | **Age at T1** | **ADI-R Social** | **ADI-R RRB** | **CBCL (AFF) T1** | **CBCL (AFF) T2** | **Age at Diagnosis** | **IQ** | **Total SCQ Score** | **ABAS CSS** |
| --- | --- | --- | --- | --- | --- | --- | --- | --- | --- |
| **Age at T1** | 1.00 | 0.10 | 0.04 | 0.12 | 0.08 | 0.00 | 0.00 | 0.09 | 0.11 |
| **ADI-R Social** | 0.10 | 1.00 | 0.12 | 0.05 | -0.07 | -**0.44** | **-0.34** | **0.66** | **-0.43** |
| **ADI-R RRB** | 0.04 | 0.12 | 1.00 | 0.17 | **0.25** | -0.21 | -0.08 | 0.17 | -0.14 |
| **CBCL (AFF) T1** | 0.12 | 0.05 | 0.17 | 1.00 | **0.60** | 0.20 | 0.13 | 0.13 | -0.19 |
| **CBCL (AFF) T2** | 0.08 | -0.07 | **0.25** | **0.60** | 1.00 | -0.03 | 0.12 | 0.01 | -0.20 |
| **Age at Diagnosis** | 0.00 | **-0.44** | -0.21 | 0.20 | -0.03 | 1.00 | **0.28** | **-0.28** | **0.27** |
| **IQ** | 0.00 | **-0.34** | -0.08 | 0.13 | 0.12 | **0.28** | 1.00 | **-0.31** | **0.48** |
| **Total SCQ Score** | 0.09 | **0.66** | 0.17 | 0.13 | 0.01 | **-0.28** | **-0.31** | 1.00 | **-0.52** |
| **ABAS CSS** | 0.11 | **-0.43** | -0.14 | -0.19 | -0.20 | **0.27** | **0.48** | **-0.52** | 1.00 |

^a^CBCL (AFF): CBCL Affective Subscale Total Score

^b^SCQ TOT: Social Communication Questionnaire Total Score

^c^ABAS CSS: Adaptive behaviour composite score.

Calculated using spearman correlation co-efficient. Bolded values p < 0.05

**
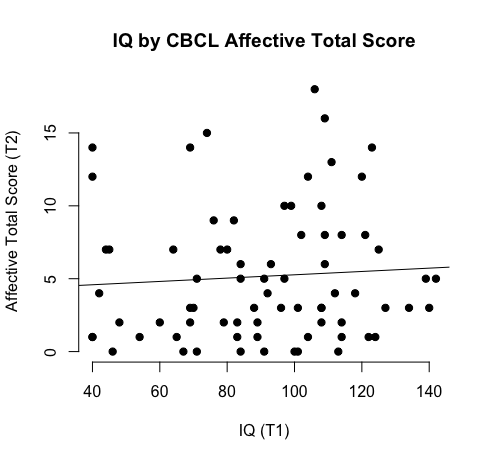
** **
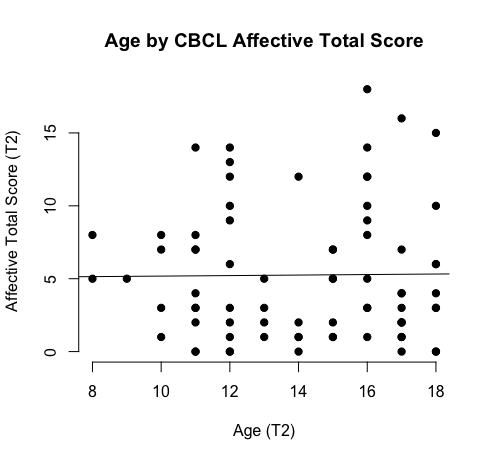
**

**
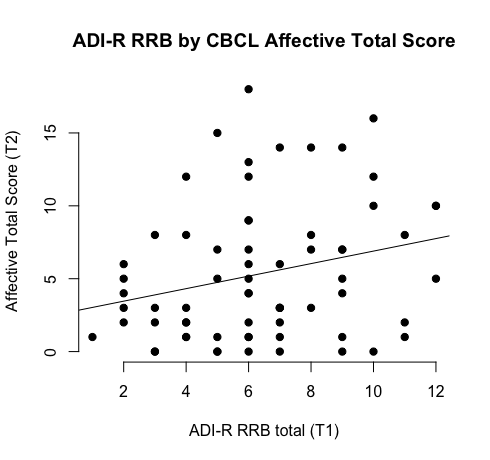
** **
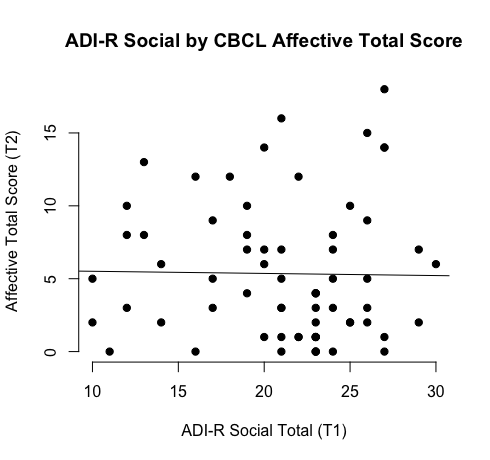
**

**
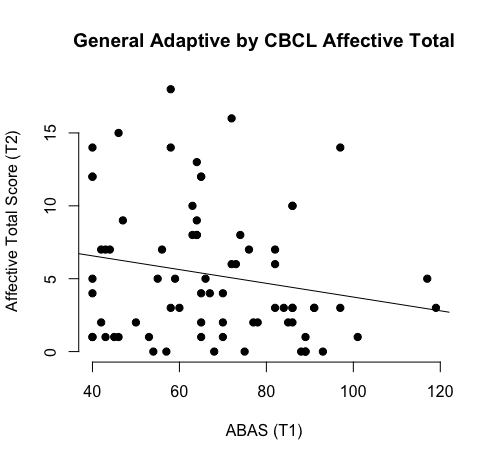
**
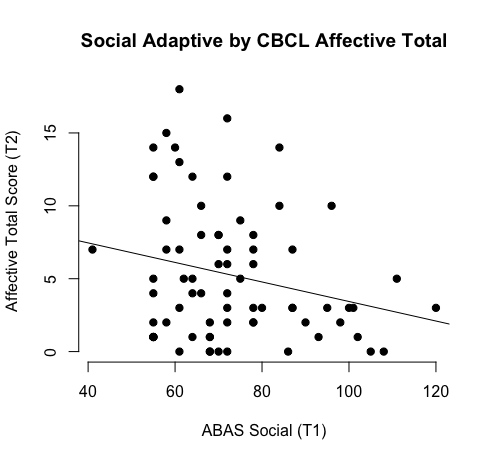


**Supplemental Figure 2: Scatter plots for variables of interest.** The y-axis denotes the CBCL Affective Problems Total score at T2 for each participant. The x-axis of each scatter plot shows a different variable of interest at T1. Variable of interests included in these analyses: IQ at T1, Participant Age, ADI-R RRB (Autism Diagnostic Interview-Revised Restricted and Repetitive Behaviour) subscale score at T1, ADI-R Social subscale score at T1, ABAS (Adaptive Behavior Assessment System) General Adaptive Composite score at T1, and ABAS Social Composite score at T1. Lines of best fit are shown to demonstrate associations between the variables of interest and the CBCL Affective Problems Total score at T2.

**Supplemental Table 2: Sensitivity Analysis Examining Odds of Clinical Elevated Depressive Symptoms at T2, adjusting for T2 age, sex, IQ and presence of depressive symptoms at T1**

| **Predictor of interest** | **aOR** | **95% CI** | **P value** |
| --- | --- | --- | --- |
| Loneliness | 3.60 | 0.98 to 13.22 | 0.05 |
| High Adaptive Skills (ABAS > 70) | 0.28 | 0.08 to 1.05 | 0.06 |
| High Social Adaptive Skills (ABAS>70) | 0.28 | 0.08 to 1.05 | 0.06 |
| High ADI-R RRB (>7) | 3.43 | 0.99 to 11.90 | 0.06 |
| Self-harm behavior | 3.04 | 0.55 to 16.80 | 0.20 |
| Suicidal Ideation | 2.67 | 0.54 to 13.10 | 0.23 |
| ADHD | 2.49 | 0.77 to 8.1 | 0.18 |
| Psychotropic medications (any) | 2.02 | 0.60 to 6.7 | 0.25 |

T1: Time point 1, baseline

T2: Time point 2, follow-up

SCQ: Social Communication Questionnaire

ABAS: Adaptive Behavior Assessment System

ADI-R RRB: Autism Diagnostic Interview-Revised Restricted and Repetitive Behaviour Subscale Score

Loneliness, self-harm, suicidal ideation were assessed on the Child Behavior Checklist. General adaptive and social skills were assessed using the Adapative Behavior Assessment System. Restricted/ Repetitive behaviours were assessed on the Autism Diagnostic Interview-Revised. Medication use refers to any psychiatric medication use by the participant at T1.

**Supplemental Table 3: Comparison between subgroups with persistent depressive symptoms (PDS) vs. non-persistent depressive symptoms**

| **Variable** | **Measure** | **Non-PDS Subgroup (N=57)** | **PDS Subgroup (N = 18)** | **Test statistic** | **P-value** |
| --- | --- | --- | --- | --- | --- |
| **Age at T1** | Mean (SD) | 9.65 (2.51) | 10.3 (3.11) | t = -0.95095, df = 73 | 0.3448 |
| **Sex** | Female (% Subgroup) | 14 (24.6%) | 4 (22.2%) | 0.88 (Fisher's exact) | 0.99 |
| **Income** | < 74,999 (%Subgroup) | 11 (19.3%) | 6 (33.3%) | X2= 0.24, DF=1 | 0.62 |
|  | > 75, 000 (%Subgroup) | 25 (43.9%) | 8 (44.4%) |  |  |
|  | No data  (%Subgroup) | 21 (36.8%) | 4 (22.2%) |  |  |
| **IQ** | Mean (SD) | 90.6 (27.4) | 92.2 (25.9) | T= -0.22429, DF= 71 | 0.82 |
|  | Median [Min, Max] | 91.0 [40.0, 142] | 102 [40.0, 125] |  |  |
|  | Missing | 1 (1.8%) | 1 (5.6%) |  |  |
| **SCQ Total score** | Mean (SD) | 19.9 (7.05) | 20.7 (7.06) | t = -0.42342, df = 72 | 0.6732 |
|  | Median [Min, Max] | 20.0 [4.00, 34.0] | 22.0 [6.00, 30.0] |  |  |
|  | Missing | 1 (1.8%) | 0 (0%) |  |  |
| **Social Skills (ABAS)** | Mean (SD) | 74.3 (17.2) | 68.1 (11.4) | t = 1.447, df = 72 | 0.1522 |
|  | Median [Min, Max] | 72.0 [41.0, 120] | 65.0 [55.0, 96.0] |  |  |
|  | Missing | 1 (1.8%) | 0 (0%) |  |  |
| **Adaptive Skills (ABAS)** | Mean (SD) | 69.4 (19.7) | 62.9 (16.8) | t = 1.2562, df = 72 | 0.2131 |
|  | Median [Min, Max] | 67.5 [40.0, 119] | 63.5 [40.0, 97.0] |  |  |
|  | Missing | 1 (1.8%) | 0 (0%) |  |  |
| **CBCL Affective Subscale T1 (Total)** | Mean (SD) | 3.56 (2.61) | 10.6 (4.26) | t = -8.4792, df = 73 | **<0.001** |
|  | Median [Min, Max] | 4.00 [0, 10.0] | 9.00 [7.00, 22.0] |  |  |
| **Psychiatric medications at T1** | Yes | 13 (22.8%) | 10 (55.6%) | W = 345 | **0.009** |
| **Family Psychiatric History** | No | 31 (54.4%) | 8 (44.4%) |  |  |
|  | Yes | 18 (31.6%) | 4 (22.2%) | 2.851e-31, df = 1 | 1 |
|  | Missing | 8 (14.0%) | 6 (33.3%) |  |  |
| **ADHD** | No | 39 (68.4%) | 7 (38.9%) | X-squared = 4.248, df = 1 | **0.04** |
|  | Yes | 17 (29.8%) | 11 (61.1%) |  |  |
|  | Missing | 1 (1.8%) | 0 (0%) |  |  |

T1: Time point 1, baseline

T2: Time point 2, follow-up

SCQ: Social Communication Questionnaire

ABAS: Adaptive Behavior Assessment System

ADIR-RRB: Autism Diagnostic Interview-Revised Restricted and Repetitive Behaviour Subscale Score

Loneliness, self-harm, suicidal ideation were assessed on the Child Behavior Checklist. General adaptive and social skills were assessed using the Adapative Behavior Assessment System. Restricted/ Repetitive behaviours were assessed on the Autism Diagnostic Interview-Revised. Family psychiatric history and medication use refers to any history of mental health conditions in first degree relatives, and any psychiatric medication use by the participant at T1.
